# Curcumin promotes skin wound healing by activating Nrf2 signaling pathways and inducing apoptosis in mice

**DOI:** 10.55730/1300-0144.5678

**Published:** 2023-09-17

**Authors:** Junli WU, Li DENG, Ling YIN, Zhirong MAO, Xiaoqing GAO

**Affiliations:** 1 Department of Human Anatomy, School of Basic Medical Sciences, Southwest Medical University, Luzhou, Sichuan, China; 2 Public Center of Experimental Technology, Southwest Medical University, Luzhou, Sichuan, China

**Keywords:** Curcumin, wound healing, oxidative stress, Nrf2, apoptosis

## Abstract

**Background/aim:**

Curcumin may have potential as a therapy for wound healing, but the underlying mechanism remains unclear. It is not known whether curcumin can promote wound healing by activating Nrf2 signaling pathway and inducing apoptosis. This study determined the role of Nrf2 signaling pathway and apoptosis in curcumin-promoting skin wound healing.

**Materials and methods:**

The full-thickness skin defect model of mice was made and randomly divided into a control group and a curcumin group. The mice in the curcumin group and in the control group received respectively a daily topical treatment of Vaseline cream with or without 5 mg curcumin. The wound healing of mice was observed daily. The mice in two groups were killed respectively on postinjury days 3, 7, and 14, and the wound tissues were collected, with 5 mice in each group. Pathological change and formation of collagen fibers were observed by HE and Masson staining respectively. The expression of caspase-3 was observed by immunohistochemistry. Western blot was used to examine the protein levels of Nrf2 and HO-1, and ELISA assay and colorimetry assay were used to check the contents of ROS, MDA, SOD, and GSH.

**Results:**

The wound healing rates of curcumin group were higher than those of control group (p < 0.05), and the pathological changes were also significantly better than those in the control group (p < 0.05). Collagen fiber synthesis in curcumin group was higher than that in control group (p < 0.05). Moreover, the expression of caspase-3 in curcumin group was higher than that in control group on 7th day post wound (p < 0.05). Furthermore, the levels of ROS and MDA in curcumin were lower than those in control group (p < 0.05), and the level of Nrf2, HO-1, SOD and GSH were higher than those in control group (p < 0.05).

**Conclusion:**

Curcumin improves skin wound healing by activating the Nrf2 signaling pathway and inducing apoptosis in mice.

## 1. Introduction

Skin is a barrier against the environment that protects the body from exogenous insults such as microbial infection, xenobiotics and ultraviolet radiation, and it is regarded as the most frequently injured tissue compared to other tissues [1]. Millions of people annually encounter skin injuries in response to surgery, trauma, skin burns, as well as chronic diseases around the world, which is a common health problem [2]. These skin injuries probably lead to significant undesired physiological and psychological influences.

Wound healing involves four temporal sequences but also overlaps basic processes including hemostasis, inflammation, proliferation, and tissue remodeling [3]. At the early stage of wound repair, immune cells recruit to the wound site and release reactive oxygen species (ROS). Low ROS levels can have a positive effect on wound healing, which resist the invasion of bacteria and other pathogens, as phagocytic neutrophils and macrophages employ their reactive and destructive properties [4–6]. Moreover, they act as secondary messengers to nonlymphoid cells and many immunocytes, regulating angiogenesis and perfusion into the wound area [5]. However, increased and prolonged levels of ROS result in oxidative stress, with delayed inflammatory reaction [7]. Nuclear factor erythroid 2-related factor 2 (Nrf2), a key factor in antioxidant stress, regulates the expression of cytoprotective genes and antioxidant substances, such as heme oxygenase 1 (HO-1), superoxide dismutase (SOD), glutathione peroxidase (GSH-Px), to scavenge free radicals [8,9]. At the late stage of wound repair, excessive inflammatory cells such as neutrophils and macrophages can be removed by apoptosis to prevent excessive inflammation, in addition, apoptosis prevents excessive scarring by removing fibroblasts and myofibroblasts [10].

Curcumin is the main natural polyphenol found in the rhizome of Curcuma longa (turmeric) and in others, Curcuma spp with multiple beneficial effects, such as antiinflammation, antioxidant, antibacterial, and anticancer [11,12], has been proven to be beneficial for wound treatment [13]. For example, Curcumin accelerated wound healing by improving rates of epithelialization and wound contraction and increasing collagen synthesis at the wound site and showed an antioxidant effect by decreasing the levels of lipid peroxides (LPs) and increasing the levels of SOD, catalase (CAT), GSH-Px [14]. Moreover, curcumin showed enhanced neoangiogenesis in the diabetic-impaired cutaneous wounds [15]. Furthermore, curcumin encapsulated in a microemulsion reduced ultraviolet radiation (UV)-induced cytotoxicity on the skin by the Keap1-Nrf2 Pathway [16].

In the present study, we investigated the mechanism of curcumin promoting wound healing by observing the effect of curcumin on the Nrf2 signaling pathway and on cell apoptosis during the early stage and late stages of wound repair.

## 2. Materials and methods

### 2.1. Animal

This study was approved by the Animal Care and Welfare Committee of the Southwest Medical University (permit number: 2020698) and was strictly performed in accordance with the Guide for the Care and Use of Laboratory Animals. A total of 35 Kunming (KM) male mice (age 8–10 weeks; weight 25–30 g) were used in this study, and were obtained from Animal Experimental Center, Southwest Medical University, Sichuan, China (license number: SYXK(Sichuan) 2018-065). All mice were housed at 23 ± 2 °C with a humidity of 60% ± 5% and a scheduled 12 h light⁄dark cycle. Throughout the experiment, animals were allowed free access sterile water and a standard laboratory mice food pellet diet.

### 2.2. Animal experiments

Mice were randomly divided into 3 groups: control group (n = 15), curcumin-treated group (n = 15), and normal group (n = 5). The control mice and curcumin-treated mice were anesthetized with intraperitoneal injection of 1% pentobarbital sodium (40 mg/kg) and the dorsal skin was cleansed with 1% iodophor and 75% ethanol. Two full-thickness excisional wounds were created in the skin on either side of the dorsal midline by excising skin and panniculus using an 8 mm diameter biopsy punch as described by Chigurupati et al [17]. Control mice were treated with 20 μL vaseline cream (Solarbio, Beijing, China, Cat NO. V8230, medical grade), and curcumin-treated mice were treated with 20 μL vaseline cream containing curcumin (Solarbio, Beijing, China, Cat NO. SC8670, HPLC≥98%) 5 mg once a day [18].

### 2.3. Wound surface-area measurements

Wound photographs were taken on day 0 (immediately after skin puncture), 3, 7, 10, and 14. Wounds were photographed. All photographs were taken by the same investigator, within the same distance of each skin wound area. All photographs were assessed using Image J v1.8.0 analysis software to measure wound surface area. Wound surface area was calculated in 3 sections per animal using the following formula: Wound surface area = (original wound area − current wound area)/original wound area × 100%.

### 2.4. Histology

The mice were sacrificed on days 3, 7, and 14 postwounding, and the skin tissues from the wound site of five mice per group were removed for histological and biochemical analyses. These samples were then separately fixed in 10% formalin, and embedded in paraffin. Serial sections of 4 μm were cut and stained with hematoxylin and eosin (H&E) (Solarbio, Beijing, China) and Masson (Jiancheng Institute of Biological Engineering, Nanjing, China) to perform respectively histological analysis and collagen formation evaluation. The sections were scanned with a digital slice scanner (Ningbo Jiangfeng Biological Information Technology Co., Ltd, Zhejiang, China). Image-pro plus 6.0 analysis software was used to calculate the collagen volume fraction in 3 sections per animal: collagen volume fraction = (collagen positive area/total tissue area) ×100%.

### 2.5. Immunohistochemical staining

Caspace-3 immunohistochemistry staining (Santa Cruz Biotechnology, USA) was used to identify cell apoptosis in wound tissue, DAB staining, and hematoxylin counterstaining. The positive cells were observed for a brownish-yellow color. Positive cells were assessed in 5 regions of interest situated within wound tissues in 3 sections per animal. Measurement was made using image-pro plus 6.0 software. The percentage of caspace-3^+^ cells was quantified by normalizing total caspace-3^+^ to the total number of cells.

### 2.6. Western blotting

Nrf2 and HO-1 protein levels were analyzed by western blotting. Briefly, total protein was extracted using lysis buffer containing 1 mM phenylmethanesulfonyl fluoride (Beyotime, Shanghai, China), measured using a BCA Protein Assay Kit (Beyotime, Shanghai, China). The proteins were denatured for 5 min at 100 °C, and then protein samples were subjected to 8%–12% sodium dodecyl sulfate–polyacrylamide gel electrophoresis and transferred to a nitrocellulose membrane (Millipore, USA). The membranes were blocked at room temperature for 2 h with 5% BSA (Sinopharm, Beijing, China) and incubated overnight with the following primary antibodies at 4 °C: anti-Nrf2 (Cell Signaling Technology, USA, 1:800), anti-HO-1 (Bioworld Technology, USA, 1:800) and anti-β-tubulin (Bimake, USA, 1: 3000). The following day, the membranes were incubated with horseradish peroxidase (HRP)-conjugated secondary antibodies (ZSGB-BIO, Beijing, China, 1: 5000) for 2 h at room temperature. Following incubation, the specific proteins were visualized with Immobilon Western Chemiluminescent HRP Substrate (Millipore, USA). β-tubulin was used as the protein loading control.

### 2.7. Enzyme-linked immunosorbent assay (ELISA) and colorimetry assay

Skin specimens were homogenized in 1 mL saline/g tissue using a glass homogenizer. The homogenates were centrifuged at 10,000 g for 30 min at 4 °C, and the supernatant was stored at −80 °C until analyzed. ROS, MDA, GSH, and SOD protein levels were determined using ELISA kits (Andy Hua tai, Beijing, China) or colorimetry kit (Andy Hua tai, Beijing, China) according to the manufacturer’s instruction.

### 2.8. Statistical analysis

All measured values were presented as mean ± SD, and statistical analysis was performed using SPSS software, version 26.0. Group differences were compared using one-way ANOVA, followed by Tukey’s post hoc tests. Difference was considered significant at < 0.05.

## 3. Results

### 3.1. Effect of curcumin on wound healing

**Figure 1:**
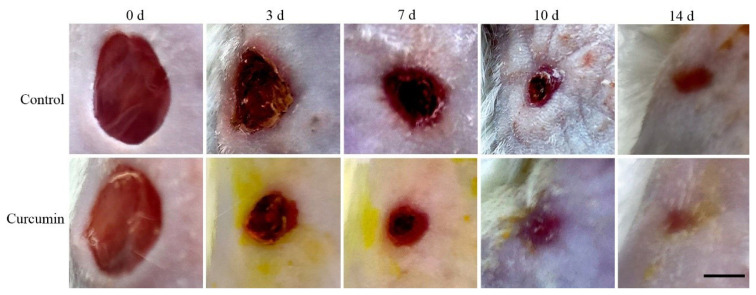
Topical application of curcumin accelerates wound healing of in mice. Images of a representative mouse from each group taken on postinjury days 0, 3, 7, 10, and 14 are shown. The full-thickness wounds were induced in vehicle- and curcumin-treated mice. Scale bar = 4 mm.

The full-thickness wounds were induced in young adult male Kunming mice and then curcumin or vehicle was applied topically to the wounds once daily. Images of the wounds were acquired on postinjury days 0, 3, 7, 10, and 14 (Figure 1), and wound sizes were quantified (Table 1). As shown in on postinjury days 3, 7, and 10 the size of wounds in curcumin-treated mice was smaller than that in control mice (p < 0.05). In control mice, the wound size decreased progressively with an average rate of wound closure of 46.96% ± 2.63% on day 3 and an average rate of 78.075% ± 7.38% on day 10. The rate of wound closure in mice treated with curcumin was significantly faster than the rate of control mice (p < 0.05), with the wounds in all curcumin-treated mice being nearly closed on day 10, and the average rate of wound closure was 95.50% ± 2.53%. On postinjury day 14, the wound had completely closed in two group, but the curcumin-treated mice had smaller scar tissue than the control mice.

### 3.2. Effect of curcumin on wound histological changes

**Figure 2:**
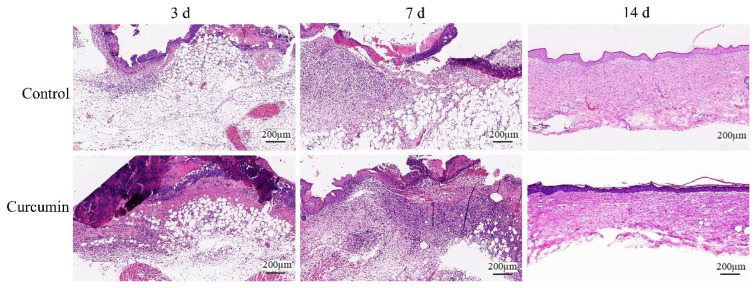
Histological features of wound healing in mice treated with curcumin or vehicle. Images of skin wound tissue sections with haematoxylin and eosin staining showing histological changes during the wound healing process in curcumin-treated mice or control mice at postinjury days 3, 7, and 14. Curcumin-treated mice exhibited enhanced restoration of epidermal and dermal tissues in the wound. Scale bar = 200 μm.

In a parallel experiment, we euthanized mice in curcumin-treated and control groups at postinjury days 3, 7, and 14 and then performed a histological evaluation of skin wound tissue sections stained with H&E. Curcumin-treated mice exhibited enhanced restoration of epidermal and dermal tissues in the wound (Figure 2). On postinjury days 3, fibrous appearance was still obvious, inflammatory cells, fibroblasts, and neo-vascularization with erythrocytes are visible but few in number in control mice. Compared with the control mice, the number of inflammatory cells, fibroblasts, and neo-vascularization are more evident in curcumin-treated mice (Figure 2). On postinjury days 7, there was a dense cell shape in two groups with more inflammatory cells, fibroblasts, new blood vessels, and moderately organized collagen, however, the above performances were more outstanding in curcumin-treated mice than those in the control mice. On postinjury days 14, the epidermis had been closed and the dermis was very well organized in comparison to day 7 in two groups. However, compared with control mice, the dermis in the curcumin-treated mice was thinner, with fewer cells and fewer new blood vessels, which was closer to the normal dermal structure (Figure 2). In addition, the epidermis of in the curcumin-treated mice was more regular than that of the control group.

### 3.3. Effect of curcumin on the growth of collagen fibers in wound tissue

**Figure 3:**
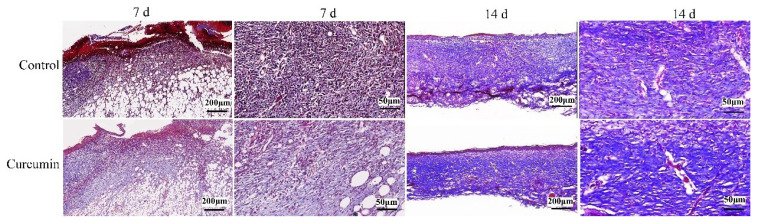
Formation of collagen fibers of skin wound tissue of mice in control group and curcumin group. Representative images of wounds on day 7 and 14 after injuring showing the contribution of curcumin to collagen volume ratio in the tissues, on 14 days, curcumin group collagen fibers were closer to normal dermal structure. Scale bar = 200 μm, 50 μm.

To further explore the promoting effect of curcumin on wound healing, we used Masson staining to evaluate the effect of curcumin on collagen fiber production in granulation tissue during wound healing. The collagen deposition in curcumin-treated mice was higher than that in control mice on postinjury days 7 and 14 (Figure 3, Table 2). Moreover, the collagen in control mice was oriented in small parallel bundles, while curcumin-treated mice presented the basket-weave collagen, which is closer to healthy dermis (Figure 3).

### 3.4. Effect of curcumin on caspase-3 expression in wound tissue

**Figure 4:**
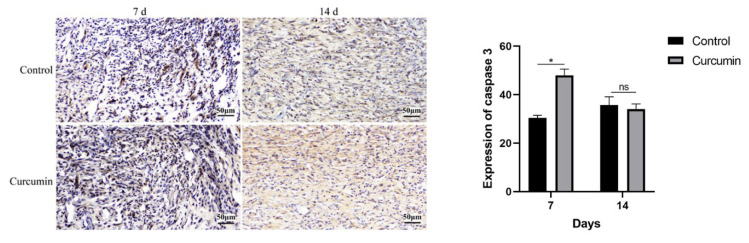
Wound histology with immunohistochemical staining. On day 7 after injury, caspase-3 expression was more significant in curcumin group than in control group, and no significant difference was observed between the two groups at day 14 (scale bar = 50 **μm). All data are representative of five independent experiments. Data were displayed as mean ±** SD (n = 5) and analyzed by ANOVA test. ^*^p < 0.05 vs. control group.

Caspase-3 is a cysteine-aspartic acid protease, which can be seen as a rather specific marker of apoptosis [19] and is known to cleave various targets and to initiate cell death [20]. Our results showed that the expression of caspase-3 in curcumin-treated mice was higher than that in control mice on postinjury days 7 (p < 0.05, Figure 4), and there had no difference between two groups on postinjury days 14 (p > 0.05), which suggested curcumin promoted the apoptosis during proliferative period of wound healing.

### 3.5. Curcumin treatment ameliorated oxidative stress in skin wound tissue

To investigate the antioxidant activity of curcumin, we analyzed oxidative stress related parameters of mice skin in two groups (Table 3). On postinjury days 3 and 7, the expressions of ROS and MDA in control mice were increased compared with normal mice (p < 0.05), while the expressions of GSH and SOD were decreased (p < 0.05). Curcumin treatment, however, significantly decreased ROS and MDA activities (p < 0.05), and increased GSH and SOD expression (p < 0.05).

### 3.6. Curcumin promotes Nrf2 and HO-1 protein expression in wound tissue

**Figure 5:**
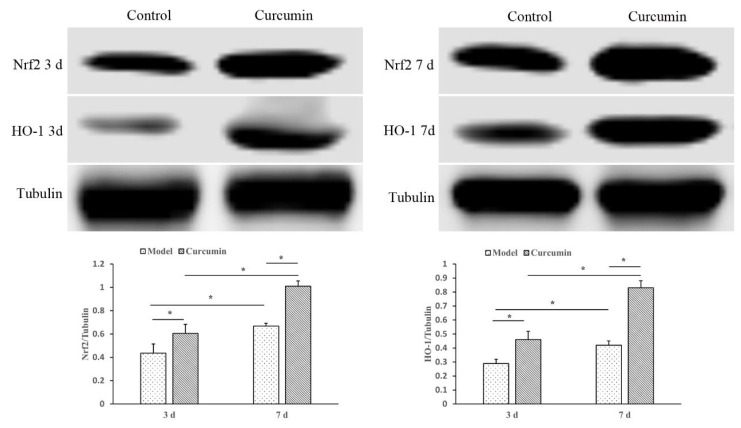
The protein expression of Nrf2 and HO-1 in skin wound tissue of mice on different days in control group and curcumin group. The protein expression of Nrf2, HO-1was analyzed by western blotting and quantitative analyses. All data are representative of five independent experiments. Data were displayed as mean ± SD (n = 5) and analyzed by ANOVA test. ^*^p < 0.05 vs. control group.

To further confirm the protective mechanism of curcumin against oxidative stress, Nrf2 and HO-1 expressions were investigated by western blotting (Figure 5). The results showed the expression levels of Nrf2 and HO-1 in curcumin-treated mice were significantly higher than those in control mice on postinjury days 3 and 7 (p < 0.05).

## 4. Discussion

Our study shows that curcumin accelerated skin wound healing, and promoted the formation of granulation tissue, collagen fiber synthesis, and cell apoptosis. Moreover, curcumin decreased the levels of oxidation products ROS and MDA and increased the expressions of antioxidants Nrf2, HO-1, SOD, and GSH in wound tissue.

In response to tissue injury, the inflammatory cells migrate to the injury site and release ROS and proinflammatory cytokines such as TNF-α, IL-1, and IL-6 [11]. ROS can damage lipids, proteins, and nucleic acids [21]. For example, products of lipid peroxidation, malondialdehyde (MDA), is a signal of the damage of ROS to the plasma membrane of cell membranes and organelles [22]. Excessive ROS in the skin promotes the activation of Nrf2 pathways [23]. Nrf2 is the major regulator of antioxidant gene expression, and during wound healing its main function is protection against the excessive accumulation of ROS. Increasing evidence indicates that overexpression of Nrf2 can mitigate cellular oxidative stress damage [23]. Studies showed that curcumin had hepatoprotective effects on HgCl2 toxicity by enhancing Nrf2-mediated HO-1 to upregulate antioxidant ability [24]. Curcumin protected rats against acute liver injury through activation of Nrf2 nuclear translocation and inhibition of NF-κB activation [25]. Moreover, curcumin caused faster and better wound healing in diabetic rats by its antiinflammatory and antioxidant potential [26]. Furthermore, curcumin indeed had powerful inhibition against damage induced by hydrogen peroxide in human keratinocytes and fibroblasts in vitro [27]. Here we showed curcumin decreased the level of ROS and MDA, and the expression of antioxidants Nrf2, HO-1, SOD, and GSH.

Of course, we also noticed that the intra-group dispersion trend of our ELISA data was a bit large in the normal and control groups, leading to a decrease in the reliability of the data. For example, the relative standard deviation (%RSD = SD/MEAN × 100%) values of the results showed that %RSD for normal group (3 d) ROS levels were 20.55%, GSH levels were 15.33%, while 10% variation within group is generally acceptable. Moreover, in the normal and control (7 d) group, the % RSD values appear higher, while the curcumin group is within limits. The reason for this result is, on the one hand, the lack of homogeneity owing to the presence of differences in sampling sites and depths. On the other hand, based on the principle of minimum sample size for animal protection, it is mainly due to the small sample size and the fact that the values with large discrete data were not removed from the statistics. The sensitivity of the test kit is also relevant. The standard deviation is the measure of dispersion, acceptability of the mean value for the group can be considered via % RSD. The mouse skin itself is very thin, and the sampling tool is limited by experimental conditions and does not employ precise methods for sampling and accurate depth quantification. However, the discrete trend of curcumin group data is small, which can explain its effect on antioxidant stress. Increasing the sample size while excluding data with large discrete trends may make the data more reliable.

The results above suggest that curcumin reduces oxidative stress damage in wound tissue by activating Nrf2/HO-1 pathway and increasing the level of antioxidant enzymes.

The proliferative phase of wound repair generally follows and overlaps with the inflammatory phase. During this time, fibroblasts migrate toward the wound area to begin the tissue-rebuilding process. Proliferating fibroblasts synthesize and secrete immature extracellular matrix (ECM) variants such as type III collagen and EDA fibronectin, as well as the type I collagen that is normally found in adult skin [28]. Various studies have shown that fibroblasts accelerate filtration into wound sites when treated with curcumin [11]. It has been found that topical curcumin accelerated wound closure with well-formed granulation tissue dominated by collagen deposition and regenerating epithelium in normal mice [29] and in diabetic rats [26]. The present study showed curcumin group had more fibroblasts and neovascularization in the dermis than the control group, with thicker and wider granulation tissue in the proliferative phase, and the collagen volume ratio in the curcumin group was higher than that in the control group. The result suggested that curcumin promoted granulation tissue formation and collagen deposition.

Reorganization of the wound tissue involves the apoptosis of a variety of cell types at the wound site and the degradation and replacement of EDA fibronectin and type III collagen with type I collagen, the organization of collagen I fibers into bundles [28]. Apoptosis is the main cause of decreasing cell numbers during the various stages of wound healing [30]. Unneeded cells, such as inflammatory cells and excessive fibroblasts in remodeling stage, are eliminated by apoptosis [30]. It has been reported that curcumin increases caspase-3 activity to contribute to the proapoptotic effect of human neutrophil apoptosis [31]. Scharstuhl A. et al. showed curcumin treatment in high doses (25 μM) induced ROS formation to cause fibroblast apoptosis in vitro, however, pretreatment with low doses curcumin (5 μM) protected against 25 μM curcumin-induced apoptosis, because preconditioning regulated HO-1 protein expression and HO-1 activity, thus protecting fibroblasts. Therefore, curcumin treatment in high doses (>25 μM) may provide a novel way to regulate pathological scar formation through the induction of fibroblast apoptosis [32]. Moreover, curcumin treatment not only increased collagen content but promoted collagen fibers also mature earlier [14]. Meanwhile, collagen fibers are more compact and well-aligned in the wound site and the bundles of collagen appeared to be thicker when curcumin was administered [33]. Here, we showed the expression of caspase 3 in the curcumin-treated group was higher than that in the control group at 7 days after injury, and on the 14th day, the collagen fibers in the curcumin-treated group were arranged in an orderly manner and in small bundles in parallel. The result suggested curcumin might promote apoptosis of various cells including inflammatory cells and fibroblasts in the proliferation phase, thus better promoting the evolution of granulation tissue to scar tissue, preventing inflammation from prolonging and reducing scar tissue.

In summary, our study indicated that curcumin can accelerate wound healing, which may be related to its ability to reduce oxidative stress, promote fibroblast proliferation, collagen synthesis, cell apoptosis and collagen fiber maturation. Therefore, topical curcumin treatment may be a good strategy to optimize wound healing.

## Figures and Tables

**Table 1:** Comparison of wound healing rates of mice in control group and curcumin group (Mean ± SD. n = 5)

Group	Day 3 (%)	Day 7 (%)	Day 10 (%)
Control	46.96 ± 2.63	62.62 ± 8.21	78.07 ± 7.38
Curcumin	58.51 ± 2.67*	86.44 ± 4.19*	95.50 ± 2.53*

* p < 0.05 vs. control group.

**Table 2:** Comparison of collagen fiber volume ratio in skin wound tissue of mice in control group and curcumin group (Mean **±** SD. n = 5)

Group	Day 7(%)	Day 14(%)
Control	18.17 **±** 3.87	66.86 **±** 4.56
Curcumin	30.17 **±** 3.36*	84.88 **±** 5.67*

* p < 0.05 vs. control group.

**Table 3:** Comparison of the levels of oxidative stress related indexes in skin wound tissue of mice in control group and curcumin group (Mean ± SD. n = 12)

	Group	ROS (U/mL)	MDA (nM/mL)	GSH (μmol/L)	SOD (U/mL)
Day 3	Normal	10.85 ± 2.23	44.16 ± 5.12	66.01 ± 10.12	40.40 ± 3.70
	Control	51.52 ± 3.21*	94.28 ± 11.17*	43.55 ± 3.18*	22.32 ± 3.91*
	Curcumin	34.54 ± 5.47#	65.20 ± 5.17#	91.70 ± 8.63#	55.01 ± 5.31#
Day 7	Control	63.35 ± 6.69*	109.76 ± 14.89*	44.03 ± 5.11*	21.83 ± 4.57*
	Curcumin	44.87 ± 4.77#	88.42 ± 7.59#	105.18 ± 10.29#	56.97 ± 9.14#

* p < 0.05 vs. normal group;

# p < 0.05 vs. control group.
